# Hopital Saint-Louis

**Published:** 1829-02

**Authors:** 


### HOPITAL SAINT-LOUIS.

*History of a rare, and perhaps unique, Case of Cancerous Tubercles in different parts of the Body.* By Dr. Lugol.

Jean Baptiste Baujoin, in his thirty-eighth year, tall, and of a robust constitution, had been affected in early childhood with tubercles in the neck, and with scaldhead, which disappeared at puberty. He married at one-and-twenty, and has had seven children, of whom only three survive. The eldest, a girl thirteen years old, has been scrofulous from her infancy, being habitually affected with ophthalmia and with tubercles in the neck.

Baujoin continued to enjoy excellent health till he was thirty-six years old: he was very strong, robust, and courageous, extremely industrious, and in the habit of carrying burthens of five hundred weight and upwards.

Being out of work in the country, he came to Paris, where he was employed in breaking up old boats and barges, in which occupation he was generally up to his knees in water, and constantly exposed to cold humidity; often passed the day, in the depth of winter, bathed in sweat, and the night in a low damp apartment, where, however, he lived pretty well, though he usually drank no wine.

Baujoin lived in this manner from the autumn of 1827 until the end of the following December, when some tubercles formed first in the left and then in the right side of the neck; almost immediately after, in the left armpit and groin, and nearly simultaneously in the corresponding regions of the right side. They were at first moveable, and rolled under the finger like small bullets, but increased in size so rapidly that in less than three months time they acquired the volume of a goose's egg.

At the same time that the tubercles reappeared in the neck and in the other regions just mentioned, the porrigo, which at puberty had spontaneously become well, again broke out at the posterior part of the scalp.

The isthmus of the fauces was much contracted by the swelling of the amygdalæ, which, as well as the arches and velum palati, were strongly pressed forward by the tubercles in the pharynx.

On the 26th April, 1828, when he came under the care of Dr. Lugol, physician to the Hôpital Saint-Louis, he had been a month without swallowing either solid or liquid food. Dr. L. tried to make him drink a few spoonsful of some very thin broth, which was soon rejected from the œsophagus; and the patient said that when liquid food, such as broth, passed through the isthmus of the fauces, it was stopped by another obstacle, on a level with the superior third of the sternum, which seemed as it were a bar beyond which it would not go through, and which mere drink could not pass but with the greatest difficulty.

His appetite was still undiminished, but this mechanical obstacle to the deglutition of food, which had continued a month, had caused great emaciaton. The limbs were wasted extremely; the flesh, where it existed, was soft; the superficial veins were prominent; the heat of the skin was of a pungent character; the pulse was at 112, rather strong; the tongue dry; bowels unmoved during the last fortnight; and the general strength of the patient was so reduced, that he could not sit up.

Baujoin, though inclined to speak, could not do so without great pain and difficulty, owing to the compression of the vocal organs. He complained of acute pain behind the middle of the sternum. The respiration could be heard in every part of the chest, yet the patient's almost inability to move, his extreme leanness, and the consequent depth of the intercostal spaces, rendered the application of the stethoscope necessarily difficult and incomplete. The respiration was less vesicular in the summit of the left than in the right lung. On feeling the belly, Dr. L. perceived in the right flank a tumor larger than two fists, which he concluded to be the liver, become tuberculous.

On the 29th April, the patient's face was swollen; his cheeks and the tumors of the neck assumed an erysipelatous appearance; his pulse was at 120; the heat was become more pungent, his depression more manifest, and he died the 30th of April, at half past five A.M.

Dr. Lugol opened the body twenty-six hours after death, in the presence of MM. Papavoine, Cuvier, Calvinhac, and Weber.

When the thorax was struck, it was observed to emit a natural sound. The aspect of the tubercles in the neck attracted the notice of Dr. Lugol the moment some of them were laid bare, when he affirmed to the pupils present that they were not of a scrofulous character, as had been believed during the patient's life, but a cancerous growth, perhaps of an unique description, and at least very rarely to be met with in the records of the profession.

As the disease of the cervical, axillary, and inguinal regions was in both sides the same, its appearance on the right side is alone described.

*Right cervical region.*—Above the centre of the jaw there was a group of tubercles, about the size of a goose's egg, situated beneath the platysma myoides, pushing upwards the tongue and muscles situated above the os hyoides. Beneath these there was a much larger assemblage of tubercles, extending obliquely in the direction of the sterno-cleido-mastoideus to within an inch of the clavicle, and reposing on the side of the pharynx. One of these last was at least as large as a hen's egg, and, with its fellow on the opposite side, had contracted the diameter of the pharynx. Beneath this second stratum a multitude of small tubercles were perceived, resembling in form and size some kidneybeans, excepting one, which was of the size of a small apricot, and which had been felt during life over the external third of the clavicle. All these collections of tubercles were connected with others which occupied the summit of the thorax, and also with those in the armpit, by small tubercles which accompanied the axillary vessels, &c.

*Right axillary region.*—In that triangular space underneath the clavicle which is included between the pectoralis minor, humerus, and clavicle, there was a tubercle, the size of a nut, which could be felt through the integuments and pectoral gland. But in the armpit there were twenty, varying from the size of a peachstone to that of a cherrystone, and two others larger than apricots. Behind the upper third of the sternum, in the anterior mediastinum, a few flat and rather small tubercles were found; and others resembling pease were seen attending the internal mammary arteries in their course downwards as far as the xiphoid cartilage.

In the history of the disease, mention has been made of the extreme difficulty which the patient experienced whenever he attempted to swallow. Besides the external pressure occasioned by the tubercles at the side of the neck, the deglutition was likewise impeded by others situated anterior to the spine; but especially by a cancerous enlargement of the tonsils, and by the growth of several tumors of the same nature at the base of the tongue. The compression of the salivary glands, the morbid growth of the tonsils and mucous glands, are to a certain degree sufficient to account for the dryness of the mouth and pharynx, and consequently for the pain occasioned by the deglutition of liquids. As to the pain which, after swallowing, was felt at the upper margin of the sternum, where a sort of bar seemed to impede the passage of the most liquid food, and even of any kind of drink, it might be attributed to the presence of three tubercles which were found encircling the corresponding portion of the œsophagus. A fourth tubercle, larger than these, and of the size of a walnut, was found on the anterior surface of the œsophagus, about an inch above its passage through the diaphragm.

In the groin there were eight large tubercles, situated below the crural arch, resembling in appearance the convolutions of the intestines, and included between Poupart's ligament, the adductors of the thigh, and the external surface of this limb. These tubercles, by means of some as small as pease, formed a communication with others which extended along the iliac artery and vein to the descending aorta. A multitude of them, of a dark red colour, and smaller than kidneybeans, were scattered over the mesentery, mesocolon, omenta, upper and lower margins of the stomach, and over the external and lateral parts of the bladder. In the right flank there was a tumor seven inches long, equally broad from right to left, and of about the same thickness. This tumor adhered above to the inferior surface of the liver, which it forcibly pushed up towards the diaphragm, the concavity of which it considerably increased; above, towards the right, and below, it adhered to the duodenum, which was stretched and carried forwards by it; on the left side, it was firmly connected by adhesions to the pancreas. The whole circumference of the tumor was composed of small round protuberances, of various sizes, which were originally perhaps so many distinct tubercles. Its colour, inferiorly and towards the right side, was a brown green, which would seem to have depended on the retarded circulation in the white vessels of the cellular membrane and contiguous parts of the peritoneum. On its anterior surface appeared the ductus communis choledocus, sufficiently distended to admit a large probe, and crossing the tumor above and behind in its course from the liver to the duodenum.

After having duly noticed the external circumstances of the tumor, Dr. L. removed it with the liver, which he easily accomplished, since it was not intimately connected with either the right hypochondrium, the spine, the vena cava, or vena porta. This tumor does not appear to have been formed in the liver, as it might have been easily separated from it, if that had been thought desirable. It had no connexions on the right side or behind; on the left, it seemed a continuation of some other tubercles that were situated under the pancreas, which they made to project forwards. Dr. Lugol suggests that this tumor owed its formation to the reunion of small tubercles, similar to those which were found in the mesentery and other folds of the peritoneum; that probably tubercles of the same character appeared along the course of the vena cava, vena porta, and hepatic vessels; and that these tubercles, small at the commencement, and variously separated from each other, had, as they increased in size, become gradually nearer together, until they at length formed the above large tumor.

In order that the interior of the tumor might be examined, Dr. L. made a longitudinal section of it precisely in the direction of its antero-posterior diameter, which measured more than six inches. The surface of this section exhibited various shades of colour, separated by certain lines, by which the boundaries of the original tubercles composing the mass might be traced, and which the lobulated surface of the tumor had already indicated before it was cut through. Amongst these tumors, the two that were situated nearest the liver were remarkable both on account of their size and whiteness, but those at a little distance from it were smaller, and presented various shades of red. At about the point where the middle and inferior thirds of this section met, was a collection of a sanguineous substance, extending more than two inches, and in all respects resembling what is found in the brain of certain individuals who have died of apoplexy.

In the left hypochondrium a large tumor was found, which proved on examination to be the spleen, double its natural size, and quite converted into an encephaloid mass. It was eight inches long, five broad, and a foot in circumference: though it retained somewhat of the original form of the spleen, it preserved no trace of the elementary structure of this organ ; it was a mere assemblage of cancerous tubercles, varying in dimensions from the size of a grain of millet to that of a nut, and differing from those of other regions by being of a deeper colour. They were in general united, intermixed, and confluent; but a few appeared to be more isolated, and as it were enveloped in a kind of cellular cyst, upon which some blood-vessels were ramified. These numerous tubercles, by their union, their intermingling, their separation, their various shades of colour, and the interstices by which they were separated from each other, produced the appearance, when divided, of rose-coloured marble, of a very singular and variegated description. This cancer of the spleen was in general of a harder consistence than those of other parts which have been already detailed.

The brain evidently contained too much fluid, blood, and serum. Its texture was also rather soft, but it was in other respects of a natural appearance. The lungs were sound, and crepitated. The heart was rather large, but could hardly be said to be *hypertrophised*. The stomach and intestines evinced various signs of congestion. The liver was pushed a little backwards, smaller than natural, and of a fine red colour, but unchanged in its structure. The kidneys, like the other abdominal viscera, were vascular, but their texture was quite sound. The blood throughout the body appeared to be suffering asphyxia within its various vessels: it abounded in the lungs, liver, kidneys, alimentary canal, and in the brain ; but it was every where black, in a fluid state, and without coagula.

Of these tumors, which were all considered during life to be scrofulous, only one contained scrofulous matter in a grumous state. All the rest were cancers, and generally of a round oval shape. They were of various sizes, and for the most part the largest were softer and more coloured than the rest: but to this there were several exceptions. By careful and minute dissection of the tubercles in the axilla, many small vessels were discovered, which could be traced back to the neighbouring arteries. As soon as these minute vessels reached the surface of a tubercle, they were subdivided into others, and spread out upon a sort of cellular texture, which formed its first envelope; then they pierced a second membrane, without subdividing again. This second coat, which was properly speaking the cyst, was somewhat thick, diaphanous, and composed of laminæ or plates of a dense texture. These minute vessels, after penetrating to the interior of the tubercle, traversed its entire surface to the very centre ; and hence the colour of these tubercles were greater or less in proportion to the number of capillaries with which they were supplied. In some cysts they were so numerous, that they formed as it were vascular tassels, which floated in the midst of the soft substance of the tubercle. In some of these tassels minute coagula of blood were seen, in some instances more dark coloured and recent than in others, and in all cases seeming to arise from rupture of these vessels. In some tubercles there were none of these vascular tassels, but a great number of distinct red filaments extended throughout their substance in every direction.

This substance itself presented several varieties. In some tubercles, in which it was of a white or reddish-white colour, it was analogous in consistence to the white substance of the fœtal brain; in others, in which it was of a deeper colour, it resembled in this particular the substance of the corpora striata.

Several of the cysts contained nothing but a sort of pus, which was either perfectly white or as transparent as an opal, and uniformly inodorous; and in those cases in which the capillary vessels penetrated to this soft matter, it was little more than putridness and corruption, resembling in colour the lees of wine.

Such was the general appearance of these cancers. But there were some in which no trace of organization existed, and in which no capillaries could be discovered, neither without nor within: others had vessels externally, which could not be traced to the substance of the cyst; and there were some tubercles whose substance was supplied with blood-vessels, though they could not be distinguished externally.

The rapid progress of this disease was certainly a very prominent feature in its character; for it is not easy to conceive how a morbid production of this nature could be so generally and so completely developed in the short space of four months. Nor is it, perhaps, less astonishing that this patient, whilst in this deplorable state, should be able to continue at work during three months, up to the period of his inability to swallow food.

The anatomical details of this case are particularly important, inasmuch as they would seem calculated to induce us to consider the nutrition of cancer to be referrible to the general laws of nutrition; and they would thus consequently tend to subvert the present prevailing opinions respecting the etiology of this disease.

According to these opinions, the structure of cancer is produced and developed by irritation, which in the first instance forms a deposit of coagulable matter. The hardening of this matter is named scirrhus; its softening, cancer, medullary sarcoma, fungus hæmatodes, accordingly as this softening is differently modified. No vessels whatever are considered to belong to the cancer, but it is said to develop itself by the juxtaposition of successive layers of this coagulable matter.

On considering this theory merely in a speculative point of view, it would be allowable to request to see this coagulable matter; to be taught the laws by which it becomes first scirrhus, and then cancer; and the source of this irritation, which is the necessary cause of every stage of the disease.

In the history of the above case, which is composed of several hundred cancers, it is manifest that they have a generic form, are furnished with cysts, on the exterior and in the interior of which small blood-vessels are abundantly distributed; so that the mode in which these cancers were nourished is evident to the senses, and clearly referrible to the commonly received laws of nutrition.

Another phenomenon, the sanguineous collection which was found in the encephaloid tumor of the abdomen, is a proof that this tumor was furnished with absorbents; since the half of this sanguineous collection contained merely the fibrine of the blood; and there is no difficulty in admitting the gradual and complete absorption of extravasated blood, as sometimes occurs in recovery from apoplexy.

Thus, then, these cancers had a form, capillary blood-vessels, white vessels (as was evinced by the action of absorption), and nerves (whose existence was demonstrated by the presence of pain); they possessed all the means of nutrition in common with other organs, and like them were endowed with an original modification, within the limits of which their development was confined.

After obtaining these anatomical data, it is still more difficult to comprehend the method which these cancers adopted in their development, in order to become first scirrhous and afterwards soft. It cannot be explained by supposing that the scirrhous condition resulted from the absorption of the fluid parts; for in that case each cancer would have diminished in size proportionably as it became harder; which is contrary to experience, since in general a scirrhus becomes at the same time harder and more voluminous, owing to some peculiar mode of nutrition inherent in itself.

M. Lugol combats the opinion of those who consider cancer not a disease *sui generis*, but only an effect of inflammation; and he thinks this view of the subject both unfounded and unsatisfactory. For by this hypothesis the difficulty is only apparently, and not really removed; since it would be still necessary to explain why inflammation is sometimes, but not usually, terminated by cancer. Besides, if cancer had an inflammatory origin, it would be cured, as most phlegmasiæ are, by an antiphlogistic treatment. If it were a local disease, the removal of the affected part, when practicable, would be an infallible remedy; and yet so many operations, when adopted for this purpose, under the most favorable circumstances, have been generally followed by such melancholy results, that the most distinguished members of the profession are henceforth ready to relinquish the attempt to exterminate cancer by the knife.

---

